# An Unusual Case of Suspected Metastatic Gastrointestinal Stromal Tumor Complicated by Streptococcus Intermedius Pyogenic Liver Disease

**DOI:** 10.7759/cureus.34397

**Published:** 2023-01-30

**Authors:** Scarlet F Louis-Jean, Giorgi Sabakhtarishvili, Amanda Damota, Maia Tavadze

**Affiliations:** 1 Medicine, Anne Arundel Medical Center, Annapolis, USA; 2 Internal Medicine, Anne Arundel Medical Center, Annapolis, USA

**Keywords:** pyogenic liver disease, streptococcus intermedius, hepatic abscess, metastasis, gastrointestinal stromal tumor

## Abstract

A gastrointestinal stromal tumor (GIST) is a mesenchymal neoplasm of the gastrointestinal tract often known to express c-KIT or platelet-derived growth factor receptor alpha (PDGFRα). Among all GI tract cancers, they account for less than 1% of cases. Most patients become symptomatic in the later stages of the tumor’s course, often presenting with insidious anemia due to gastrointestinal bleeding and metastasis. The recommended management of solitary GIST is surgery, while larger or metastatic tumors that express c-KIT are managed with imatinib as either neoadjuvant or adjuvant therapy. Due to the progression of these tumors, they are at times associated with systemic anaerobic infection, which is an indication of malignancy workup. In this case report, we discuss a 35-year-old woman who was discovered to have GIST with possible hepatic metastasis complicated by pyogenic liver disease due to S*treptococcus intermedius* and the diagnostic challenge of differentiating between infection and tumor.

## Introduction

A gastrointestinal stromal tumor (GIST) is one of the most common pleomorphic non-epithelial KIT- or platelet-derived growth factor receptor alpha (PDGFRα)-mediated mesenchymal tumors of the gastrointestinal (GI) system, and they account for less than 1% of all GI tumors [1]. GIST can occur at any age, but most patients are over 40 years old, with a median age of detection of 65 years [2]. There is a similar rate of incidence between genders, with a slight propensity among men [3]. The most common location for GIST is the stomach (55.6%), followed by the small bowel (31.86%), colorectum (6%), esophagus (<1%), and very rarely extra-gastrointestinal systems [4]. Clinical presentation is variable, and patients can be asymptomatic in 18% of cases with incidental identification of GIST [5].

## Case presentation

A 35-year-old woman with a history of genital herpes on valacyclovir presented to the emergency department (ED) due to sharp 10/10 left upper quadrant (LUQ) pain, fever, and loss of appetite. On presentation, she was febrile with a temperature of 101 °F and tachycardic with a heart rate of 108. The physical exam was notable for an ill-appearing woman with severe discomfort due to abdominal pain and LUQ tenderness on palpation. Her initial laboratory results showed severe anemia with hemoglobin 5.6 g/dL requiring a transfusion of 2 units of packed red blood cells, a white blood cell count (WBC) of 11.2 × 103/µL, sodium 130 mmol/L, and elevated alanine transaminase (ALT) of 101 IU/L and aspartate transaminase (AST) of 115 U/L.

Following the initial stabilization of her hemoglobin, the patient spent the majority of her early hospital course reporting persistent LUQ pain unchanged in intensity. She underwent CT abdomen and pelvis (A/P) without contrast, which revealed a left upper quadrant nonspecific 5.5 cm × 4.2 cm mass containing a small amount of fluid and air, multiple unquantified hepatic lesions, the largest measuring 3.3 cm × 4.8 cm, which was suspicious for metastatic disease, splenomegaly with splenic infarcts, and possible masses of the pancreas or porta hepatis. Cancer antigen 125 was mildly elevated at 37 U/mL (reference range <35 U/mL), cancer antigen 19-9 was 6 U/mL (reference range <34 U/mL), and carcinoembryonic antigen was 1.7 ng/mL (reference range 0-2.5 ng/mL). The patient subsequently underwent a CT-guided liver biopsy for presumed metastatic disease by interventional radiology, with 5 mL of purulent fluid extracted and sent for culture. Preliminary results demonstrated rare Gram-positive cocci which speciated to Streptococcus intermedius. Her anaerobic blood cultures also contained Streptococcus intermedius. The echocardiogram was negative for valvular vegetation.

**Figure 1:**
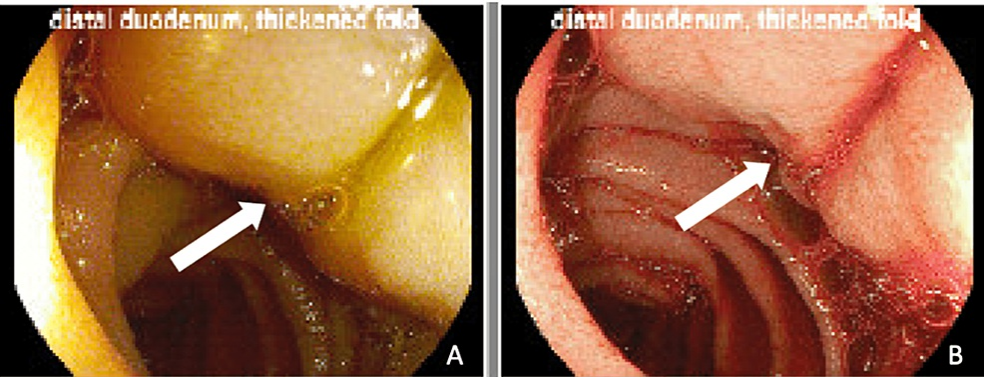
Esophagoduodenoscopy demonstrating a mass in the third portion of the duodenum, observed as an edematous mucosal wall indicated by the arrowheads under natural light (A) and narrow band imaging (B).

**Figure 2:**
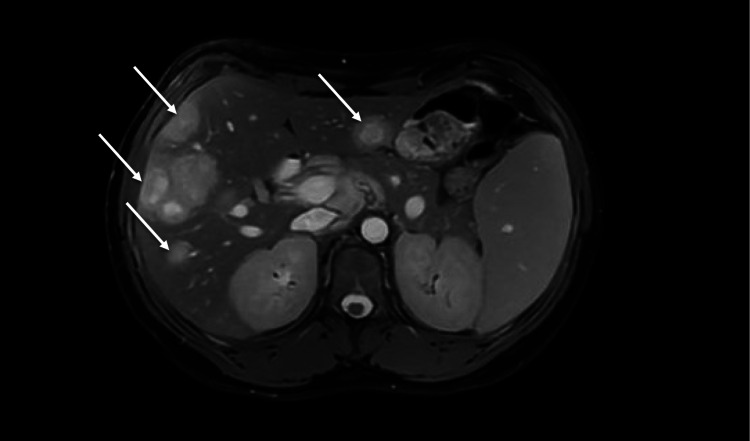
MRI of the liver with and without contrast demonstrating several variable sized lesions in the liver with a nonspecific appearance, representing either multiple abscesses or necrotic metastases (demarcated by arrows).

The patient was initially started on cefepime, then switched to piperacillin-tazobactam, and later to ceftriaxone due to an unimproved clinical course and pending culture data. Following the speciation of her anaerobic culture, a peripherally inserted central catheter (PICC) line was placed, and the patient was switched to ertapenem, which led to a significant improvement in her leukocytosis. However, a repeat CT A/P performed seven days later, showed no marked improvement in the sizes of her lesions despite antibiotics, with the largest lesion approximating 5.8 cm × 4.6 cm. She then underwent esophagoduodenoscopy (EGD) and colonoscopy. While the latter was unremarkable, the former revealed a nodular mass in the third portion of the duodenum, from which a biopsy was obtained (Figure 1). An MRI of the abdomen was subsequently performed for better characterization and revealed a partially cystic soft tissue mass in the left mid abdomen measuring 3.6 cm × 4.4 cm × 4.8 cm, along with at least 24 variably sized lesions in the liver with a nonspecific appearance believed to represent either multiple abscesses or necrotic metastases (Figure 2). Additional drainage of these masses was deferred due to their multiplicity and proximity to the diaphragm, which raised concerns for potential iatrogenic diaphragmatic perforation. 

**Figure 3:**
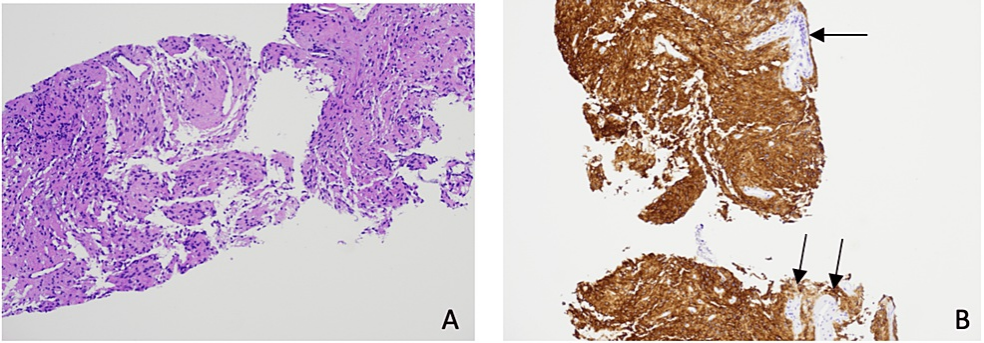
The first image (A) is an H&E stain showing spindle cell proliferation. The second image (B) shows diffuse and strong brown CD117 and focally positive CD34 (indicated by arrows) immunohistochemical staining, and negative stains for smooth muscle, actin, and desmin.

The patient’s genetic mutational analysis of her biopsied mass revealed very low levels of the c-KIT mutation on exon 9 [c.1504_1509dup (p.A502_Y503dup)] and negative mutations of D816V on exon 17 and PDGFRα. Immunohistochemical staining was notable for the presence of CD117, patchy staining for CD34, and negative staining for actin and desmin, which was consistent with GIST (Figure 3). Additionally, the stromal cells demonstrated bland cytologic morphology and absent mitotic activity. The patient was then scheduled to follow up with hematology/oncology for the initiation of imatinib 400 mg daily and with infectious disease for the continued management of her antibiotics, of which she completed a total of 32 days. Palliative care was also initiated, and the patient was discharged with opiate therapy for the management of persistent and severe abdominal pain. The patient, however, was lost to follow up with our institution as she transitioned her care to a healthcare facility closer to home.

## Discussion

GIST is the preferred term used for mesenchymal tumors localized specifically to the GI tract and are differentiated from other mesenchymal neoplasms, such as leiomyomas, leiomyosarcomas, schwannomas, and neurofibromas, based on their expression of the proto-oncogene tyrosine-protein kinase kit (c-KIT), tyrosine kinase receptor CD117, CD34, and sometimes actin [6,7]. Approximately 20% of GIST may not stain for biomarkers of smooth muscle, and smaller percentages of the tumor do not harbor a KIT or PDGFRα mutation [6,8]. Those that do harbor KIT mutations most commonly involve exon 11 (70%) and exon 9 (10-15%) [8]. Exon 9 mutations have an observed association with small intestinal and colonic tumors [8].

GIST is considered potentially malignant and is stratified from very low to high based on its clinical risk of malignancy and recurrence [9]. Fletcher et al., in 2002, created the National Institute of Health (NIH) classification system that stratified the tumor into very low, low, intermediate, and high-risk for recurrence based on size and mitotic activity [10]. In 2006, Miettinen et al. developed the Armed Forces Institute of Pathology (AFIP) classification, which examined anatomical location in addition to tumor size and mitotic rate [11]. Then, in 2008, Joensuu et al. created the modified NIH, which found that tumor rupture confers the highest risk of recurrence [12]. No classification strategy, however, has proven superior to another [5].

GIST has been hypothesized to closely simulate the interstitial cells of Cajal (the pacemakers of the gut) or their precursors, but they are being increasingly recognized as arising from mesenchymal cells with multipotent properties [2]. The most common locations of GIST, in decreasing order of prevalence, include the stomach, small intestine, colon, rectum, and esophagus [3]. There are cases of GIST occurring in extra-gastrointestinal organs, known as EGIST, which less commonly occur in the mesentery, retroperitoneum, omentum, gallbladder, pancreas, liver, and urinary bladder [3]. Approximately one-third of GISTs are incidentally detected via imaging or surgical procedures, but the most common clinical symptoms of the neoplasm include GI bleeding, which can be chronic, insidious anemia, or life-threatening hematemesis [1]. This is owed to the high vascularity of the tumor [2]. Additional physical manifestations include gastric discomfort, tachycardia, abdominal pain, fullness, or bowel obstruction [2]. Approximately 70% of patients are symptomatic [3]. In 20% of cases, patients are found to have metastases at the time of the diagnosis, with most involving the peritoneal cavity and the liver, with rare occurrences involving the lungs, bones, or brain [1,2]. The five-year survival rate of patients with metastatic GIST is thought to be around 55% [13].

Based on the pathobiology and molecular cascades involved in the development of GIST, an accurate histologic diagnosis and prognostication are essential before initiating treatment [14]. GIST sampling is performed via endoscopic mucosal biopsy, endoscopic ultrasound-guided fine needle biopsies, tru-cut histologic biopsies, and CT-guided needle core biopsies, the latter of which is recommended for larger tumors [1]. The pathologic diagnosis is based on the identification of spindle cells or epithelioid histology, and in the case of small intestinal GIST, additional common features include extracellular collagen globules and neuropil-like material [1]. GISTs are known to be resistant to standard chemotherapy, thus a mutational analysis to determine the presence of KIT and PDGFRα is needed to appropriately initiate tyrosine kinase inhibitors, such as imatinib, the chemotherapy shown to increase progression-free survival and overall survival in patients with non-resectable GIST [2]. Our patient, unfortunately, had a questionable tumor burden complicated by potentially numerous hepatic abscesses, which made surgical resection a nonviable option. In her case, she qualified for treatment with imatinib based on her c-KIT positivity and consistent immunohistochemical staining. Additionally, given her tumor size, location, absence of mitotic activity, and lack of established tumor rupture, she was stratified as having a low risk of recurrence according to each of the risk stratification tools described above, although she had an overall poor prognosis due to suspected metastasis.

Imatinib is used to control advanced GIST via neoadjuvant uses for reducing tumor size and facilitating resection, and as adjuvant therapy to reduce recurrence in patients who are at high risk [15]. Imatinib improves survival time and delays disease progression in many patients and operates by interrupting the downstream signaling cascade involved in cellular proliferation [15]. Drug responsiveness has been shown to lead to a partial response in 65-70% of patients, with only 5% having a complete response, with a median duration exceeding two years [15]. Fifteen to twenty percent, however, develop stable disease, while 10-20% experience resistance [15]. KIT mutations on exon 11 have higher response rates to imatinib therapy than those with exon 9 mutations, which are the strongest prognostic risk factors for disease progression and death [16]. PDGFRα is generally responsive to imatinib [16]. The optimal imatinib dose recommended for initial therapy is 400 mg daily, which was determined by two-phase III trials conducted with patients with advanced GIST [16]. However, it has been shown that tumors harboring an exon 9 mutation may fare better with 800 mg/day, although the rate of toxicity and side effects are higher with increased doses [16]. In our patient’s case, oncology opted for the initial standard dose due to the complexity of her case, mild transaminitis, and concerns for the consequent rampancy of her Streptococcus intermedius pyogenic infection.

A review of the literature has demonstrated that primary liver abscesses when associated with primary GIST are often caused by commensal, obligate, or facultative anaerobic organisms [17]. Alpha hemolytic streptococcus infections, which are part of the mucosal flora of the GI tract, such as Streptococcus milleri and Streptococcus intermedius, have been implicated [17,18]. While there are limited data on liver abscesses and primary GIST, common causes of pyogenic liver abscesses are related to hepatic malignancy, biliary tract disease, and gut microbiota dysbiosis [17]. The postulated mechanisms by which GISTs cause bacterial infections include systemic invasion via mucosal breakdown by the tumor or concurrent travel of the bacteria through a vehicular-mediated mechanism involving tumor translocation [14]. Emerging studies have also noted a relationship between gut microbiota dysbiosis and the development of GIST, which we would recommend further exploration [19]. Nonetheless, if there is a concern for liver metastasis, it is recommended that patients undergo diagnostic workup for pyogenic liver abscesses as well, so they are promptly initiated on appropriate antibiotics [18]. Conversely, the identification of an alpha-hemolytic streptococcus infection or, generally, an anaerobic infection should prompt further evaluation of GI tract malignancies [18]. Our patient was discharged on ertapenem via PICC line; however, due to the unknown ratio of hepatic abscess to tumor burden, a specified course of antibiotics could not be defined. Additionally, IV antibiotics alone, without further abscess drainage, were presumed to make the control of her infection difficult.

All in all, this case was demonstrably a diagnostic challenge, as we did not know whether the patient truly had metastases, although she was recommended to initiate treatment with imatinib. She was unfortunately lost to follow up with our institution as she transitioned her care to a facility closer to home. Nevertheless, despite her degree of diagnostic uncertainty, we recommend that pharmacotherapy be appropriately guided to manage both infection and malignancy, although there is no literature suggesting appropriate dosing or an antibiotic course in cases such as this.

## Conclusions

GIST is a mesenchymal tumor that can be found incidentally at advanced stages, often presenting with chronic, insidious anemia. The disease can also be associated with anaerobic infections involving alpha-hemolytic streptococci, which, if encountered first, should warrant workup for gastrointestinal malignancy. The treatment of GIST is often surgical for tumors that are deemed resectable; however, in non-resectable or advanced tumors, imatinib is the first-line treatment in patients with c-KIT mutations. In cases where patients present similarly to those described in this case report, we recommend that treatment be focused on managing both presenting problems.
